# Monitoring Nrf2/ARE Pathway Activity with a New Zebrafish Reporter System

**DOI:** 10.3390/ijms24076804

**Published:** 2023-04-06

**Authors:** Lorenzo Badenetti, Rosa Manzoli, Michela Rubin, Giorgio Cozza, Enrico Moro

**Affiliations:** 1 Department of Women’s and Children’s Health, University of Padova, I-35128 Padova, Italy; 2 Pediatric Research Institute “Città della Speranza”, I-35127 Padova, Italy; 3 Department of Molecular Medicine, University of Padova, I-35121 Padova, Italy; 4 Department of Biology, University of Padova, I-35121 Padova, Italy

**Keywords:** Nrf2, zebrafish, transgenic reporter, drug

## Abstract

Among multiple cytoprotective mechanisms, eukaryotic cells exhibit a complex transcriptional program relying on the Nrf2 transcription factor, which is generally recruited upon biological stressors including oxidative-stress-based cellular insults. The relevance of this master regulator has remarkably emerged in recent years in several research fields such as cancer, inflammatory disorders and age-related neurological diseases. Here, we document the generation and characterization of a novel Nrf2/ARE pathway biosensor fish which exhibits a dynamic spatiotemporal expression profile during the early developmental stages. The transgenic line is responsive to known Nrf2 pathway modulators but also to Edaravone, which direct activity on the Nrf2 pathway has never been documented in a live transgenic fish model. We also show that the reporter is faithfully activated during fin regeneration, and its degree of expression is slightly affected in a glucocerebrosidase (Gba1) morphant zebrafish model. Therefore, this novel transgenic fish may represent a valuable tool to be exploited for the characterization of zebrafish models of human diseases, as well as for primary high-throughput drug screening.

## 1. Introduction

During their life, cells experience many potential mechanical, physical and chemical insults that may undermine their survival eventually leading to death. In multicellular organisms, oxidative stress, which is induced by metabolic and environmental agents, exposes cells to potentially progressive damage that often results in the onset of inflammation. Particularly, the chronic loss of cellular redox balance underlies the dysregulation of the immune system and the activation of pro-inflammatory cascades [1]. In non-pathological conditions, cells under oxidative stress react by inducing an antioxidant response that is able to rebalance their redox state and prevent the detrimental effects of reactive oxygen species (ROS). One master regulator of this antioxidant response is the nuclear factor erythroid 2-related factor 2 (NRF2), which was discovered in 1994 [2]. Under basal physiological conditions, NRF2 is kept at low levels due to its ubiquitination and degradation mediated by Kelch-like ECH-associated protein 1 (KEAP1), an adaptor component of a Cullin3-based ubiquitin E3 ligase complex [3]. In an unbalanced cellular redox state, when ROS levels are overproduced, NRF2 detaches from KEAP1 and translocates into the nucleus, where it heterodimerizes with one of the small musculoaponeurotic fibrosarcoma (MAF) oncogene homolog proteins, forming a transcriptional complex. This multi-protein complex is able to bind target responsive elements (called AREs, antioxidant response elements) in the regulatory regions of genes coding for proteins involved in redox balancing, cell metabolism, detoxification, stress response and iron metabolism [4]. Since their discovery in late 1980s, ARE sequences have been exploited for the generation of reporter constructs and in vitro/in vivo detection of oxidative stress induction [5,6,7,8], as well as for performing analyses of the Nrf2/ARE pathway modulation in pathological conditions [9,10]. Since then, no other novel in vivo Nrf2/ARE reporter transgenic systems have been created. In this work, we describe the development and characterization of a new Nrf2/ARE reporter transgenic zebrafish line. We provide a description of its early developmental expression pattern and faithful responsiveness to known Nrf2 pathway modulators (agonists and antagonists), including the recently approved FDA drugs Omaveloxolone (RTA-408) and Edaravone (MCI-186). We also demonstrate, in agreement with results from a previous investigation [11], that the reporter expression levels are slightly dysregulated in a Gba1 morphant zebrafish model. Using a fin amputation model, we finally provide evidence that reporter activation is triggered upon injury, suggesting its potential application for regeneration studies.

## 2. Results

### 2.1. Dynamic Spatiotemporal Reporter Expression in the Transgenic Line Tg(8XAORE:EGFP)^ia201^

**Figure 1:**
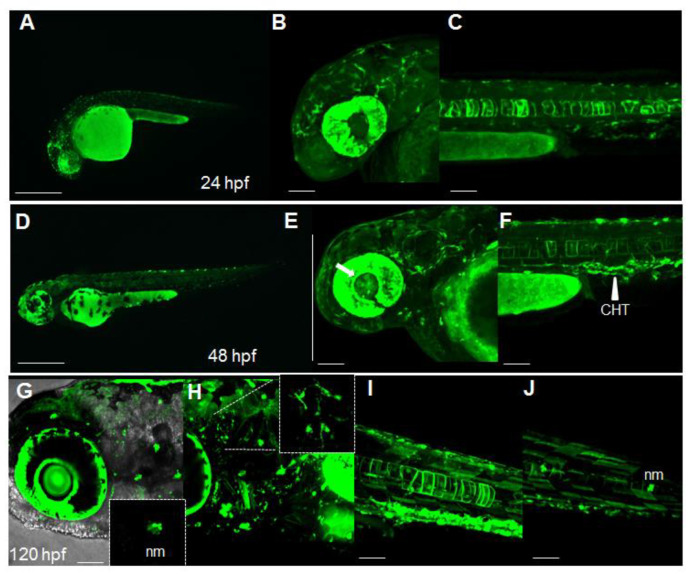
Dynamic spatiotemporal reporter expression in the Nrf2/ARE transgenic line. (**A**) Whole-mount fluorescence microscopy of a *Tg(8xAORE:EGFP)^ia201^* larva 24 hpf, showing the widespread expression of reporter-expressing cells in both cephalic and more caudal regions. (**B**,**C**) Confocal Z-stack projection of the cephalic area (**B**) and trunk region (**C**) of a 24 hpf transgenic larva in which strong fluorescent cells are detectable in the brain, eye and notochord. (**D**) Whole-mount fluorescence microscopy of a 48 hpf *Tg(8xAORE:EGFP)^ia201^* larva, showing the prolonged expression of the reporter in the cephalic and trunk regions. (**E**,**F**) Confocal Z-stack projection of the cephalic area (**E**) and trunk regions (**F**) of the same 48 hpf transgenic larva exhibiting novel expression domains in the lens and inner eye (white arrow) and the caudal hematopoietic tissue (CHT) (white arrowhead). (**G**–**J**) Confocal Z-stack projection of the cephalic area (**G**,**H**) and trunk regions (**I**,**J**) of a 120 hpf transgenic larva showing fluorescent neuromasts (nm) and protrusion-carrying brain cells (small inset in **H**), as well as muscle fibers along the trunk (**I**,**J**). All images are lateral views with anterior to the left. Scale bar in (**A**,**D**): 500 μm; in (**B**,**C**,**E**,**F**) and (**G**–**J**): 100 μm.

While searching for candidate Nrf2/ARE-responsive elements in the promoter of zebrafish genes and according to previously reported predicted consensus sequences [5], we noticed that the TGAG/CNNNGC sequence was shared among ARE elements of Homo sapiens, Mus musculus and Danio rerio. We therefore assumed that a previously published Nrf2 reporter system, which harbors eight tandem repeated ARE sequences (5′-GTGACAAAGCA-3′) derived from the mouse and rat Glutathione-S-Transferase Alpha (Gsta) promoters [6], could also be used in zebrafish. We isolated the eight cis-elements from the original plasmid pGL3-8xARE and cloned them into the 5′entry vector of the Tol2 toolkit [12]. After R4/R3 recombination with an EGFP-carrying middle entry vector, a polyA-SV40 3′ entry vector and the Tol2pA destination vector, we microinjected the final destination pDest*(8xAORE:EGFP)* vector into one-cell-stage embryos. Several independent founders were identified from the offspring obtained by outcrossing the microinjected fish with wild-type mates. All the offspring derived from different founders displayed a comparable fluorescence in the same body areas, ruling out potential genomic positional and variegation effects. In stable *Tg(8xAORE:EGFP)^ia201^* fish, fluorescence was barely detectable in the earliest developmental stages (from 8 to 20 h post fertilization, hpf), while from 24 hpf, discrete fluorescent regions were discernible in several body areas including eyes, brain, and notochord (Figure 1A-C). At 48 hpf, novel fluorescent domains were detectable, such as lens (Figure 1E, white arrow), dendritic cells of the skin, trunk blood vessels and the caudal hematopoietic tissue (CHT; Figure 1F, white arrowhead). Using confocal microscopy reconstructions of 120 hpf larvae, we were able to locate several fluorescent positive neuromasts (nm, small inset on the bottom of Figure 1G), fluorescent protrusion-bearing cells in the brain (Figure 1H, small inset on the top, and Video S1). A strong fluorescence was also traceable in the gut regions, in muscle fibers along the trunk and in more caudal regions (Figure 1J). At 7 days post-fertilization (dpf), we also detected reporter fluorescence in motoneurons and in the heart and an increased number of fluorescent muscle fibers along the trunk (Figure S1 and Video S2). Therefore, we could conclude that in our novel transgenic line *Tg(8xAORE:EGFP)^ia201^*, shortly designated Nrf2/ARE, reporter activity is dynamically detectable in several tissues and cell populations during the first developmental stages.

### 2.2. The Tg(8xAORE:EGFP)^ia201^ Line Is Responsive to Nrf2 Pathway Modulators

To verify whether the newly generated transgenic line is a *bona fide* Nrf2 pathway reporter, we performed several pharmacological tests based on the administration of well-known Nrf2 pathway agonists and antagonists. Toward this aim, we provided 8 hpf transgenic larvae with RTA-408 (Omaveloxone), a semi-synthetic oleanane triterpenoid with anti-inflammatory activity and pro-regenerative potential that is currently under clinical investigation in phase II clinical trials [13,14]. We also treated age-matched larvae at 8 hpf with the quassinoid Brusatol, which is isolated from the *Brucea javanica* plant and exhibits a sensitizing effect of cancer cells to chemotherapeutic drugs [15].

**Figure 2:**
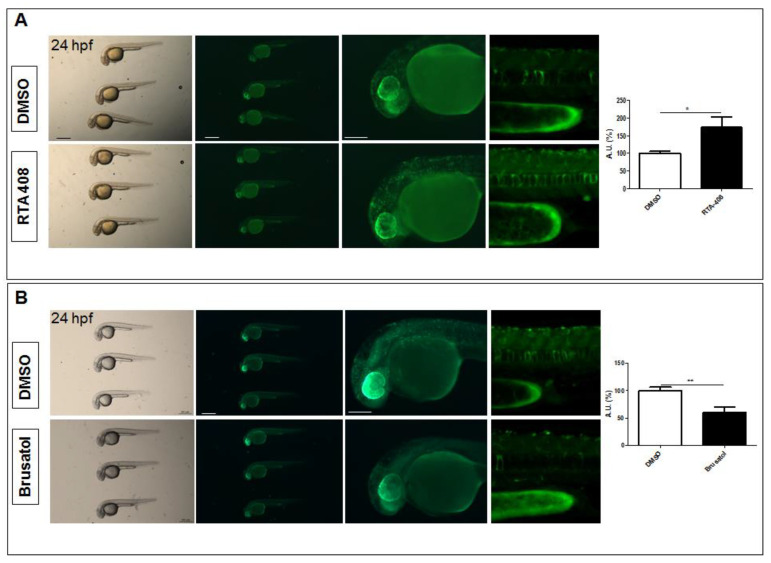
Pharmacological validation of Nrf2/ARE reporter fish. (**A**) Whole-mount bright field and fluorescence microscopy acquisition of a 24 hpf transgenic larva treated with DMSO and the Nrf2 pathway agonist, RTA-408, for 16 h. A significant increase in fluorescent cells is detected in RTA-408-treated larvae when compared to DMSO-treated fish. (**B**) Whole-mount bright field and fluorescence microscopy acquisition of a 24 hpf transgenic larva treated with DMSO and the Nrf2 pathway antagonist, Brusatol, for 16 h. A visible decrease in reporter fluorescent intensity is visible in Brusatol-treated larvae. All images are lateral views with anterior to the left. The magnifications of the trunk regions are confocal Z-stack acquisitions. The graphs reported on the right depict the ImageJ-based quantification of the selected trunk region of 5 independently treated fish (* *p* < 0.05; ** *p* < 0.005, *t*-test). Scale bars in the panels with multiple fish: 500 μm; scale bars on the panels with a single magnified larva: 100 μm.

As shown in Figure 2A,B, the Nrf2 transgenic reporter fish demonstrated increased and decreased fluorescence after treatment with the RTA-408 and Brusatol, respectively. The effects of these two drugs were also consistently maintained after 24 and 48 h of treatment, although differences were less evident (Figure S2A,B). We also tested the known Nrf2 agonist dimethyl fumarate (DMF) [16] in 24 hpf transgenic larvae for 24 h and observed increased reporter activity in all treated fish (Figure S3A). We then provided 8 hpf reporter fish with the small molecule ML-385, which inhibits Nrf2 DNA binding [17], but we found that ML-385 inconsistently reduced reporter activity in the treated fish. Collectively, according to these data, we could conclude that the *Tg(8xAORE:EGFP)^ia201^* line is sensitive to some of the well-known Nrf2 pathway agonists and antagonists, and the changes induced by these drugs are coherent with its function as a potential Nrf2 pathway readout.

### 2.3. The Nrf2 Reporter Transgenics Serve as a Platform for the Identification of Nrf2 Pathway-Mediated Biological Activity of Small Molecules

**Figure 3:**
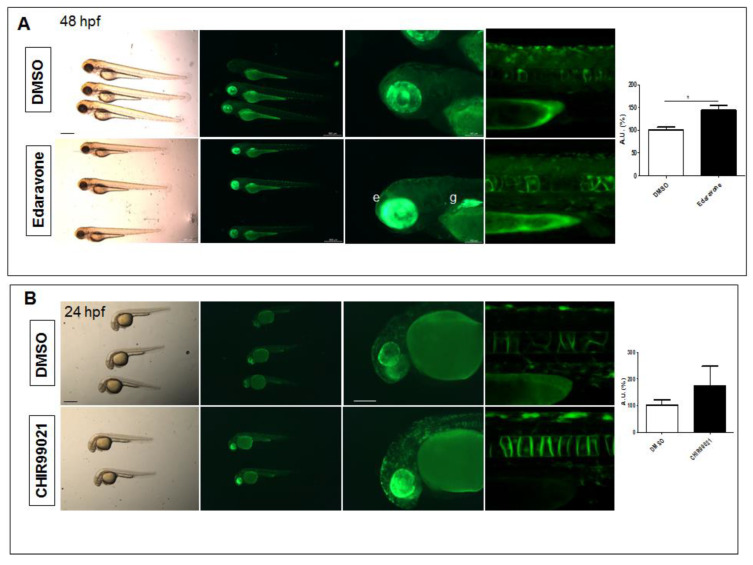
Pharmacological testing of Nrf2/ARE reporter fish. (**A**) Whole-mount bright field and fluorescence microscopy acquisition of 48 hpf transgenic larvae treated with DMSO or the radical scavenger Edaravone for 24 h. An evident increase in fluorescence is detected in the eye (e) and gut (g) of Edaravone-treated larvae when compared to DMSO-treated control fish. (**B**) Whole-mount bright field and fluorescence microscopy acquisition of 24 hpf transgenic larvae treated with DMSO or the GSK3β antagonist, CHIR99021, for 16 h. Increased reporter fluorescence is observed throughout the whole larvae when compared to the DMSO-treated control fish. The magnifications of the trunk regions are confocal Z-stack acquisitions. The graphs reported on the right depict the ImageJ-based quantification of the selected trunk region of 5 independent treated fish. (* *p* < 0.05, *t*-test) All images are lateral views with anterior to the left. Scale bars in the panels with multiple fish: 500 μm; scale bars on the panels with a single magnified larva: 100 μm.

To assess whether the Nrf2 reporter line could be exploited for the detection of Nrf2 pathway modulation by additional small molecules, we provided the transgenic fish with the known radical scavenger Edaravone (MCI-186), which has been already shown to dampen the progression of amyotrophic lateral sclerosis (ALS) and exert antioxidant effects in asthma and cerebral infarction through the Nrf2 pathway [18,19]. By treating 8 hpf fish for 16 h (Figure 3A) or 48 h (Figure S4A), we detected a mean 1.4-fold increase of fluorescence, with a particular evident increase in the eye and gut and ectopic expression of the reporter EGFP. We also treated 8 hpf transgenic fish with the Glycogen Synthase Kinase 3 Beta (GSK3β) inhibitor CHIR99021 for 16 h (Figure 3B) and observed a mean 1.7-fold reporter activity increase at 24 hpf, although we measured an inter-individual variability in fluorescence and no evident differences at 48 hpf (Figure S4B). Therefore, we could infer that Nrf2 reporter fish are sensitive to drug manipulation to a different extent according to the small molecule used and the treatment duration.

### 2.4. Beta Glucocerebrosidase Knockdown Does Not Significantly Affect Nrf2 Reporter Activation but Induces the Upregulation of a Restricted Number of Nrf2 Target Genes

**Figure 4:**
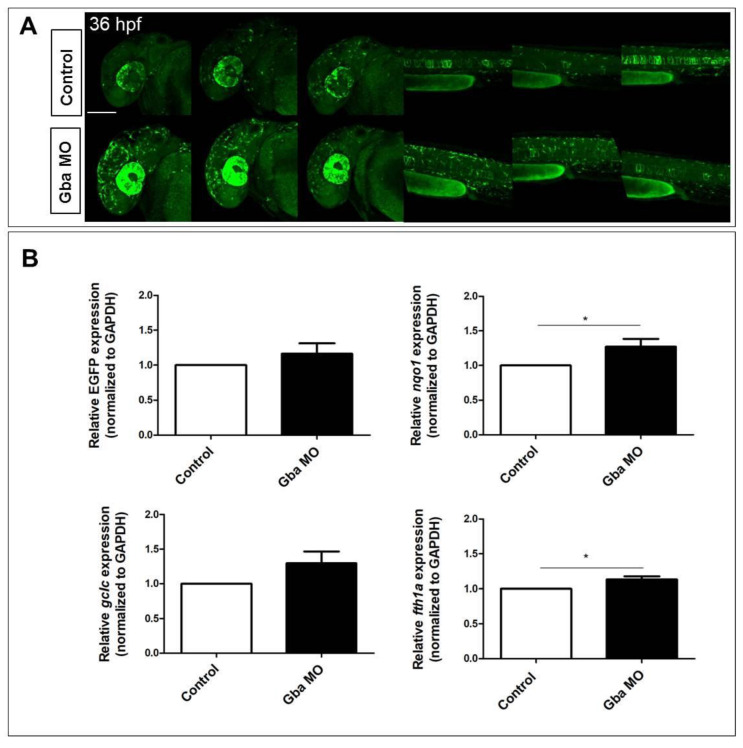
Beta glucocerebrosidase knockdown does not significantly increase Nrf2/ARE reporter expression but does induce the upregulation of Nrf2-pathway-related targets. (**A**) Confocal Z-stack projections of 30 hpf control and Gba1 morphant fish, showing increased reporter expression in both cephalic and caudal regions of morphants. For comparison, three different morphant and control fish are depicted. All images are lateral view with anterior to the left. Scale bar: 100 μm. (**B**) Bar graphs showing the quantitative EGFP reporter and Nrf2 pathway-related genes expression measured in morphant and control fish RNA extracts. Data are mean ± SD of three independent biological replicates (each replicate consists of ten larvae per condition; * *p* < 0.05; *t*-test).

We previously reported that the knockdown of Gba1 with antisense morpholinos induces an early oxidative stress response in zebrafish larvae [11]. To verify whether the Nrf2/ARE pathway could be modulated by Gba1 knockdown, we microinjected the previously used Gba1 morpholino in one-cell stage *Tg(8xAORE:EGFP)^ia201^* embryos and performed confocal microscopy analyses on the morphants and age-matched controls. As shown in Figure 4A, we observed a variable degree of ectopic expression of the reporter transgene in 36 hpf morphants. We then performed a quantitative analysis of the reporter (EGFP) and the known Nrf2 target genes’ mRNAs in microinjected fish and age-matched controls via RQ-PCR (Figure 4B). While we could identify an apparent increase in morphants for all tested markers, we measured only a significant difference for the nitric oxidase (*nqo1*) and ferritin heavy polypeptide 1a (*fth1a*) mRNAs in morphants when compared to age-matched controls. Therefore, we could conclude that while Gba1 knockdown was not able to substantially increase the Nrf2/ARE reporter activation, it significantly induced the expression of a restricted number of Nrf2 pathway-related target genes.

### 2.5. Nrf2/ARE Pathway Is Induced upon Fin Amputation

**Figure 5:**
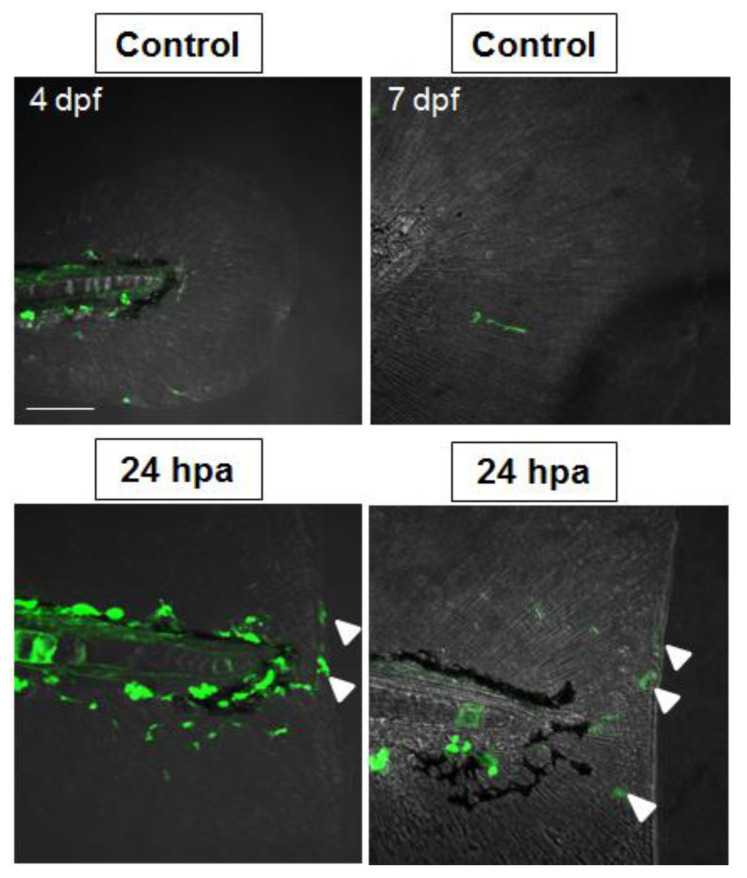
Nrf2/ARE pathway activity increases in regenerating tail fins. Representative Z-stack projection of a confocal fluorescence microscopy acquisition of unamputated and amputated tail fin of representative 4 dpf and 7 dpf Nrf2/ARE transgenic fish after at 24 h post amputation (hpa), showing fluorescent cells migrating along the stump (white arrowheads). Scale bar: 100 μm.

Considering the role of antioxidant response during larval fish fin amputation [20] and since the Nrf2/ARE pathway has been shown to play a key role during diabetic wound regeneration [21], we tested whether fin amputation in *Tg(8XAORE:EGFP)^ia201^* larvae could affect reporter expression. Toward this aim, we performed fin amputation in 3 dpf and 6 dpf transgenic larvae and monitored reporter activity changes by confocal microscopy. As shown in Figure 5, after 24 h post amputation (hpa) we could detect increased reporter activity in the wounded area, with protrusion-bearing fluorescent cells lining or moving along the amputated region. During a time-lapse recording, the same cells interacted by their protrusions (Figure S5 and Video S3). By performing the same fin clipping in double *Tg(mpeg:mCherry)^ump2^/Tg(8XAORE:EGFP)^ia201^* fish and in double *Tg(LysC: DsRED)^nz50^/Tg(8XAORE:EGFP)^ia201^*, we were able to rule out that the same fluorescent cells were macrophages (Figure S6). Therefore, we could conclude that fin amputation in Nrf2 transgenics increases the recruitment of reporter-expressing cells in the wounded area.

## 3. Discussion

When exposed to distinct biological stressors, including environmental insults and oxidative stress, cells quickly respond with the activation of a complex cytoprotective program based on the expression of genes involved in drug detoxification (phase I, II and III enzymes), glutathione (GSH) metabolism and redox homeostasis [22]. Among the potential molecular hubs of this coordinated program, the master transcription factor Nrf2 has received significant attention in the latest two decades [4].

To dynamically trace its related pathway activity (Nrf2/ARE), several tools have been generated, including chimeric vectors that harbor a tandem array of synthetic ARE elements upstream of a luciferase coding sequence for use in vitro or in vivo analyses [6,8,23]. However, in these experimental systems, the qualitative and quantitative detection of the Nrf2/ARE pathway activity by luciferase assays often required laborious approaches and dedicated live imaging equipment with limited resolution. Our group and others have previously shown that live reporter zebrafish represent a valuable alternative model in which the combination of fish optical transparency with the use of synthetic transgenes allows for the fine tracing of target cellular pathway modulation [24,25]. In particular, we already provided evidence that chimeric constructs containing synthetic cell-signaling responsive elements and a minimal promoter (human, mouse or even lower-vertebrate-related) can be integrated into the zebrafish genome, allowing for the generation of faithful and highly sensitive transgenic reporter lines [24,26]. In this work we describe the characterization of a novel Nrf2/ARE reporter fish line which, according to the preliminary data provided, may represent a novel living model to detect the modulation of the Nrf2 pathway in space and time. A previous zebrafish Nrf2/ARE reporter model exhibited limited sensitivity since low levels of mCherry expression could be detected only from 2 dpf [10]. Indeed, our newly generated Nrf2/ARE reporter fish exhibit a high and tunable expression of the reporter (EGFP) in several tissues, including the eye, brain, gut, muscle, notochord, caudal hematopoietic tissue (CHT), heart and blood vessels, already from 24 hpf. While for some of the reporter-expressing tissues, including the gut and CHT, the involvement of Nrf2 pathway could be inferred from previous studies [10,27,28], the identification of Nrf2/ARE pathway activation in the notochord and heart was unexpected. Indeed, notochord formation is known to be finely regulated by Notch signaling [29], and ARE elements have been found upstream of the Notch1 major transcription site [30], suggesting that notochordal expression of the reporter may be indirectly associated to Notch signaling developmental modulation by the Nrf2 pathway. Regarding the heart, while extensive literature has provided evidence of the Nrf2 pathway role in cardiac remodeling [31], a direct proof of its activation during the early developmental stages has not yet bee provided. To establish the pharmacological responsiveness of the Nrf2/ARE transgenics, we tested known Nrf2 pathway antagonists (ML385, Brusatol) and agonists (RTA-408), including the fumaric acid ester dimethylfumarate [32], which was already able to induce the activation of the reporter at an early developmental stage. We next preliminarily evaluated the effects on the reporter of the small molecule CHIR99021 (Laduviglusib), a known aminopyrimidine GSK3β inhibitor which selectively competes for the ATP binding site of GSK3β [33]. The activation of the reporter activity by CHIR99021 may be consistent with its inhibitory role on KEAP1-independent Nrf2 proteasomal degradation [34]. However, the detected inter-individual variability among fish prevented us from confirming the Nrf2 reporter responsiveness to CHIR99021 treatment. We also included in our preliminarily pharmacological tests the small molecule Edaravone, which has been recently under clinical evaluation for amyotrophic lateral sclerosis (ALS) (Clinical trial:NCT04569084). The induction of reporter activity in our transgenic line fits well with the knowledge that the drug acts through the Nrf2 pathway by unknown mechanisms [35].

To verify whether any loss of gene function could impact the Nrf2 pathway activity, we tested the transgenic line in a previously characterized glucocerebrosidase morphant zebrafish model [11]. Through previous transcriptomic analyses and assays, we demonstrated that the Gba1 morphants exhibit increased oxidative stress induction and a significant decrease in *gpx1b* expression [11]. Increased oxidative stress with decreased *Gpx1b* mRNA levels was already detected in a conditional knockout mouse model for the selenocysteine tRNA gene [36]. Although the increase was not statistically significant for the reporter EGFP and the target gene *gclc*, our data point to a likely increase in the Nrf2 pathway activation in the zebrafish Gba1 loss-of-function model. The lack of a significant quantitative increase in the reporter EGFP might be due to the limited sensitiveness of RQ-PCRs on RNAs derived from whole-embryo lysates. Alternatively, the extent of Nrf2/ARE pathway modulation by Gba1 knockdown could be restricted to only few tissue domains. Further tests will be required to confirm the former or the latter hypothesis.

We finally demonstrated that caudal fin amputation during larval stages triggers the activation of the reporter transgene expression in the wounded area. In particular, we observed that protrusion-carrying reporter-expressing cells were migrating and populating the same area. Using two independent, macrophage-related transgenic lines crossed with our reporter, we could rule out that these cells were indeed macrophages. Since the *Tg(LysC: DsRED)^nz50^* also labels a subpopulation of neutrophils [37], we suspect that the protrusion-bearing cells might be a subpopulation of mature neutrophils recruited during the regeneration process, but future investigations will enable to clarify this aspect.

In conclusion, in this work, we provide a preliminary description and characterization of a new Nrf2/ARE transgenic model. While further extensive tests using genetic models will be required to prove the robustness of this new reporter line, we envisage that our novel transgenic fish will provide an alternative tool for Nrf2 pathway modulation in high-throughput drug screenings.

## 4. Materials and Methods

### 4.1. Generation Tg(8XAORE:EGFP)^ia201^ Fish

A cassette containing eight multimerized copies of the antioxidant responsive elements (ARE) (5′-GTGACAAAGCA-3′), derived from the mouse glutathione S-transferase alpha 1 (*Gsta1*), was retrieved by KpnI-BglII digestion of the pGL-8XARE-lux plasmid kindly provided by Prof. Roland Wolf (University of Dundee, Scotland). The 135 bp fragment containing the ARE elements was next cloned into the Tol2-derived 5′-entry vector p5E-MCS (http://tol2kit.genetics.utah.edu/index.php/P5E-MCS accessed on 16 June 2021) digested with the KpnI-BamHI. The 8xARE-containing 5′entry vector was finally subjected to R4-R3 recombination, together with the pME-EGFP and p3E-polyA vectors, into the final destination vector pDestTol2pA [12].

A total of 250 pgs/embryo of Tol2 recombinant plasmid were coinjected with 350 pgs/embryo of in vitro synthesized transposase mRNA into one-cell stage zebrafish embryos. Microinjected embryos were raised to adulthood and outcrossed with wild-type fish. Almost 50% of the screened fish were identified as founders using an M165FCF1 dissecting microscope (Leica, Milan, Italy).

### 4.2. Drug Treatments

For drug screening tests, 8 hpf or 24 hpf embryos were treated for 12 and 24 h with 5 μM ML-385, 500 nM Brusatol, 10 μM dimethyl fumarate (DMF), 2 μg/mL Edaravone (MCI-186) (Merck, Milan, Italy), 500 nM Omaveloxolone (RTA-408) (Medchemexpress, Dba, Milan, Italy) and 5 μM CHIR99021 (Merck, Milan, Italy). The drugs were directly added to the fish water in 24-well plates. For each treatment performed in triplicates, at least 10 embryos were used.

### 4.3. Morpholino Injection, RNA Extraction and RQ-PCR

Gba1 functional knockdown was obtained using the previously described morpholinos (11). The MOs were dissolved in Danieau’s buffer (58 mM NaCl, 0.7 KCl, 0.4 mM MgSO_4_, 0.6 mM Ca(NO_3_)_2_ and 5 mM HEPES pH 7.6). Before the injection, MOs were denatured at 65 °C to avoid the formation of aggregates. Embryos at the one-cell stage were injected with 10 nl of a solution containing 12.5 ng/embryo of either targeting morpholino or its mismatched control. At 36 hpf, the microinjected embryos were collected and homogenized in Trizol reagent (Thermofisher, Monza, Italy), and the total RNA was isolated using the standard Trizol-chloroform-ethanol extraction procedure. RNA were resuspended in 20 μL of RNAse-free water. RNA sample concentrations and purity were measured by Nanodrop2000c (Thermofisher, Monza, Italy). A total of 2 μg of RNA was reverse-transcribed into cDNA using a SuperScript III Reverse Transcriptase (Thermofisher, Monza, Italy), according to standard procedures. The cDNA was subsequently subjected to SYBR Green-based real-time PCR using a RotorGene 3000 (Qiagen, Milan, Italy) and amplified with the oligonucleotides reported in Table S1. RT-PCR data were analyzed using a manually set threshold, and the baseline was set automatically to obtain the threshold cycle (Ct) value for each target. GAPDH was used as an endogenous housekeeping control gene for normalization. Relative gene expression among samples was determined using the comparative Ct method (2^−ΔΔCt^). Results are expressed as the mean relative expression ± SD of three independent replicates.

### 4.4. Tail Fin Amputation

Fin amputations of zebrafish 3 days and 6 days post fertilization (dpf) were performed as previously described (26). Following amputations, the fish were placed back in the fish water, and after 4 h or 24 h post amputation (hpa), they were anesthetized with 0.03% Tricaine and immobilized on 1.5% low-melting agarose-containing dish prior to confocal microscopy acquisition.

To verify the identity of Nrf2-reporter-expressing cells in the caudal fin region, we performed the fin clipping and confocal analyses on double *Tg(mpeg:mCherryF)^ump2^/Tg(8XAORE:EGFP)^ia201^* and *Tg(LysC:DsRED)^nz50^/Tg(8XAORE:EGFP)^ia201^*. The *Tg(mpeg:mCherryF)^ump2^* was kindly provided by G. Lutfalla.

### 4.5. Image Acquisition and Processing

For confocal microscopy, the PTU-treated larvae were embedded on 1.5% low-melting agarose and placed on a Petri capsule filled with fish water. Confocal stacks were recorded, using a low-intensity laser (20% intensity) to minimize laser-induced cell damage and photobleaching. A Nikon C2 confocal system using a 20X or 40X immersion objective (Nikon, Torino, Italy) was used. For time lapse imaging, a 1-h recording with a pause every 5 min was carried out. All images were finally analyzed with ImageJ 1.53t software (http://rsb.info.nih.gov/ij/ accessed on 12 October 2022).

## Data Availability

Not applicable.

## Supplementary Materials

The following supporting information can be downloaded at: https://www.mdpi.com/article/10.3390/ijms24076804/s1.
