# Supplementary material for: Monitoring Nrf2/ARE Pathway Activity with a New Zebrafish Reporter System
Source: Int J Mol Sci. 2023 Apr 6;24(7):6804. doi: 10.3390/ijms24076804 (PMC10095123; doi:10.3390/ijms24076804)
Supplement: Supplementary file 1 [file ijms-24-06804-s001.zip › ijms-2221055-Supplemental Data (Table S1- Figure S1-S6).pdf]

Target Gene	Primer sequence		Sequence reference
EGFP	Forward	5′-ACGTAAACGGCCACAAGTTCA-3′	[12]
	Reverse	5′-AAGTCGTGCTGCTTCATGTG-3′	[12]
Zf-gclc	Forward	5′-AAGAAACATGCTGACCACGT -3′	ENSDART00000017148.8
	Reverse	5′-CCAACATGTACTCCACCTCG-3′	
Zf- nqo1	Forward	5′-GAGAGTTTGCCGATTCACCA-3′	ENSDART00000004964.8
	Reverse	5′-ACGCTTCTCAATGAACTGCC	
ZF-fth1a	Forward	5′-CGCGAACATGCTGAGAAACT -3′	ENSDART00000027329.8
	Reverse	5′-TTTCTCCAGCTGCAGGGC-3′	

Table S1. List and sequence of primers for RQ-PCR tests.

Supplementary Figures

Figure S1. Expression of the Nrf2/ARE reporter at 7 dpf**.** (A-B) Representative confocal Z-stack projection of the cardiac region with a magnification (B), showing a visible expression of the reporter in the heart and heart valves. (C-D) Confocal Z-stack projections of the trunk region of a 7 dpf larva showing the localized expression of the reporter in motoneurons (mn), blood vessels (bv) and muscle fibers (D). Note the presence in C of a fluorescent positive dendritic cell on the top, and the persistent expression of the reporter in notochordal cells. All images are lateral view with anterior to the left. Scale bar: 100 µm.

Figure S2. Prolonged pharmacological response of the reporter to the agonist RTA-408**.** (A, B) Representative whole-mount bright field and fluorescent images of Nrf2/ARE reporter fish treated for 24 or 48 hours with RTA-408, starting the treatment at 24 hpf. Increased fluorescence is detectable in the eye, brain and notochord of RTA-408 treated fish. All images are lateral view with anterior to the left. Scale bar: 100 µm.

Figure S3. Tuning *in vivo* reporter activity by Nrf2 pathway modulators**.** (A) Representative whole-mount bright field and fluorescent images of Nrf2/ARE reporter fish treated for 24 hours with DMF starting the treatment at 24 hpf. Increased reporter activity is seen throughout the whole larva. The bar-graph on the right depicts the ImageJ-based analysis of fluorescence detected in 5 independent larvae per condition (B) Representative whole-mount bright field and fluorescent images of Nrf2/ARE reporter fish treated for 16 hours with ML-385 starting the treatment at 8 hpf. A clear decrease of fluorescent reporter expressing cells is visible in both cephalic and caudal regions of ML-385 treated larva. All images are lateral view with anterior to the left. Scale bar: 100 µm.

Figure S4. Modulation of Nrf2 reporter activity by small molecules. (A) Representative whole-mount bright field and fluorescent images of Nrf2/ARE reporter fish treated for 48 hours with Edaravone starting the treatment at 24 hpf. Increased reporter activity is visible particularly in the eye, gut and trunk regions. (B) Representative whole-mount bright field and fluorescent images of Nrf2/ARE reporter fish treated for 48 hours with CHIR99021 starting the treatment at 8 hpf. No evident changes of reporter expression are detectable in CHIR99021-treated larvae when compared to DMSO-treated fish. All images are lateral view with anterior to the left. Scale bar: 100 µm.

Figure S5. "Touch and kiss" of protrusion-bearing cells in the wounded caudal fin region of Nrf2/ARE transgenics after fin amputation. Time lapse-recording showing two reporter expressing cells (one with a visible fluorescent nucleus) getting in contact at the wounded area and extending cytoplasmic protrusions towards each other. All images are lateral view with anterior to the left. Scale bar: 50 µm.

Figure S6. Reporter-expressing cells in the wounded caudal fin are not macrophages. Representative confocal Z-stack projections of the amputated caudal fin region of 4 dpf Tg*(LysC:DsRED)*^{nz50}/ Tg*(8XAORE:EGFP)*^{ia201} *and 7 dpf* Tg*(mpeg:mCherry)*^{ump2}/Tg*(8XAORE:EGFP)*^{ia201} fish, showing no evident colocalization of *mpeg*-mcherry and *lysC*-DsRed expressing cells with Nrf2/ARE-reporter expressing cells after 24 hours post-amputation (24 hpa). All images are lateral view with anterior to the left. Scale bar: 50 µm.

Video S1. 3D-confocal reconstruction of the cephalic region of a 5 dpf Nrf2/ARE reporter fish.

Video S2. Confocal Z-stack sequence of the trunk region of a 7 dpf Nrf2/ARE reporter fish.

Video S3. Time lapse recording related to Figure S5. Each frame was recorded every 5 minutes.
