# Supplementary figures and images for: Monitoring Nrf2/ARE Pathway Activity with a New Zebrafish Reporter System
Source: Int J Mol Sci. 2023 Apr 6;24(7):6804. doi: 10.3390/ijms24076804 (PMC10095123; doi:10.3390/ijms24076804)

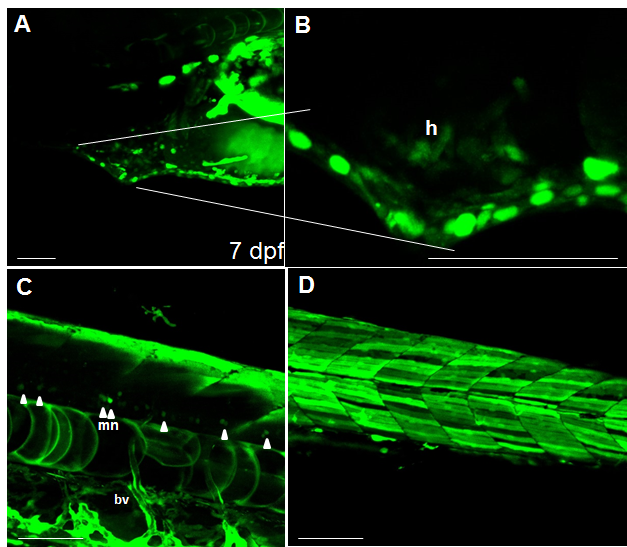

Supplement: Supplementary file 1 [file ijms-24-06804-s001.zip › Figure S1.tif]

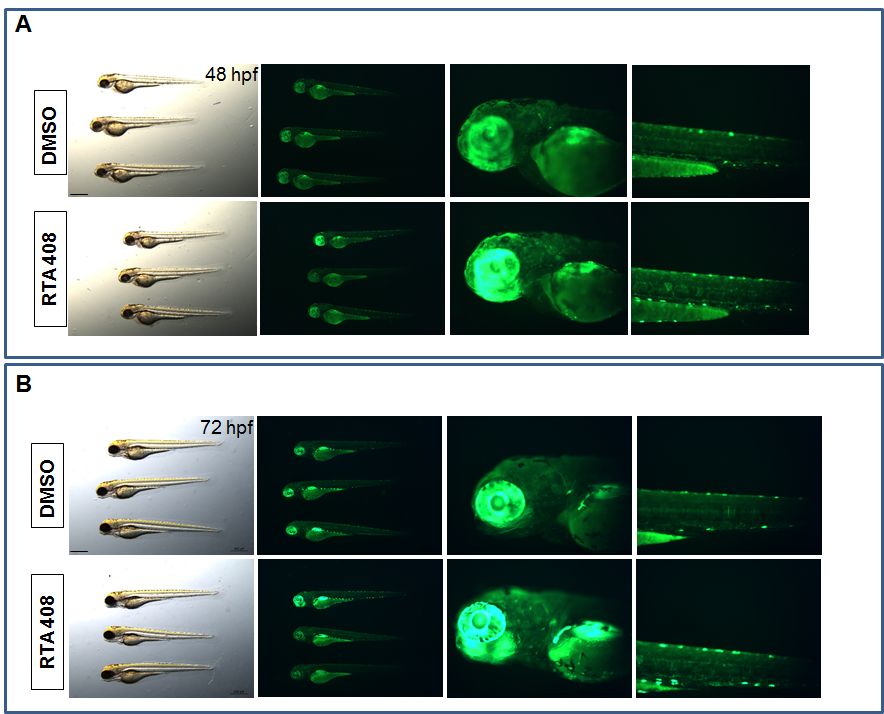

Supplement: Supplementary file 1 [file ijms-24-06804-s001.zip › Figure S2.tif]

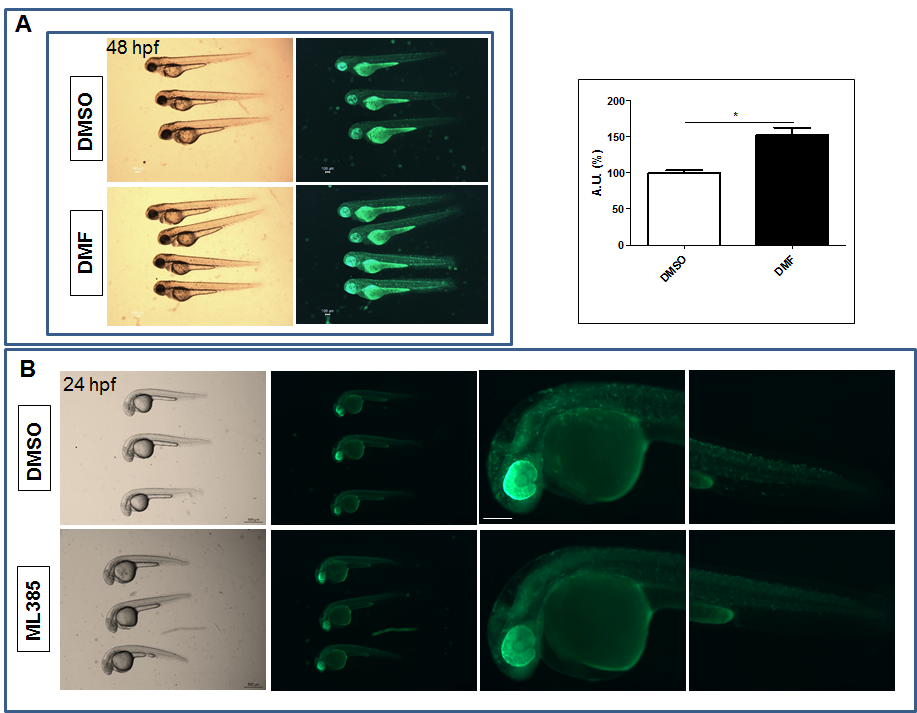

Supplement: Supplementary file 1 [file ijms-24-06804-s001.zip › Figure S3.tif]

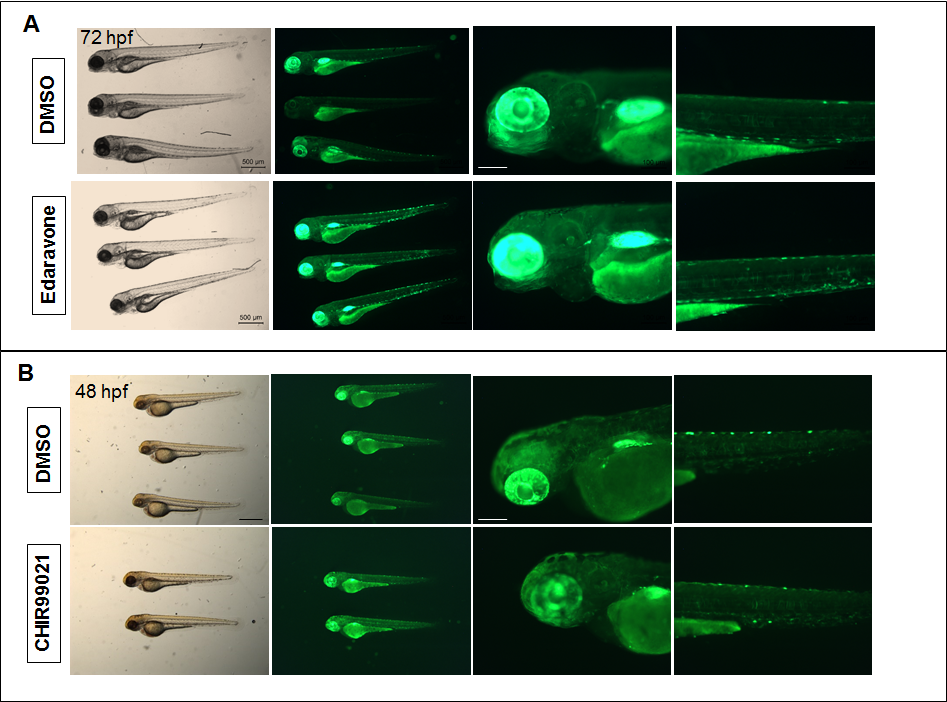

Supplement: Supplementary file 1 [file ijms-24-06804-s001.zip › Figure S4.tif]

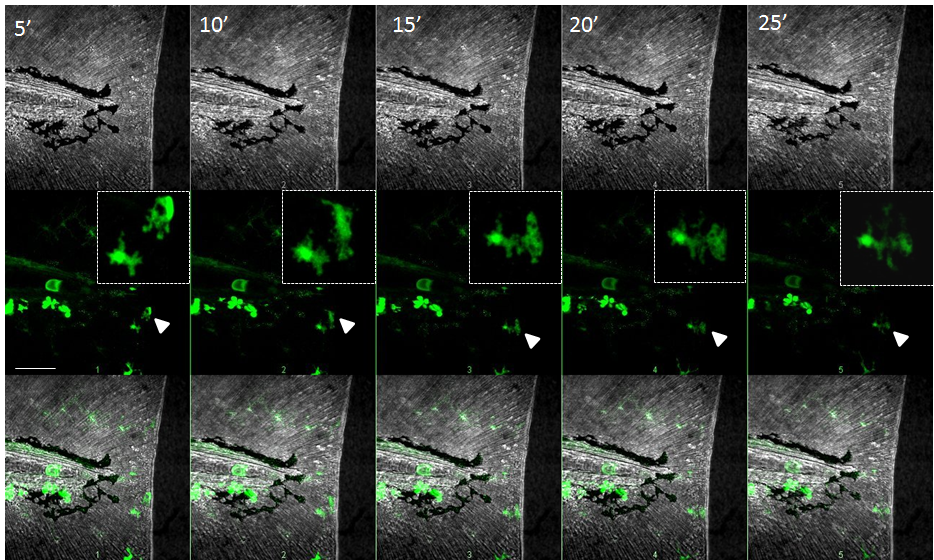

Supplement: Supplementary file 1 [file ijms-24-06804-s001.zip › Figure S5.tif]

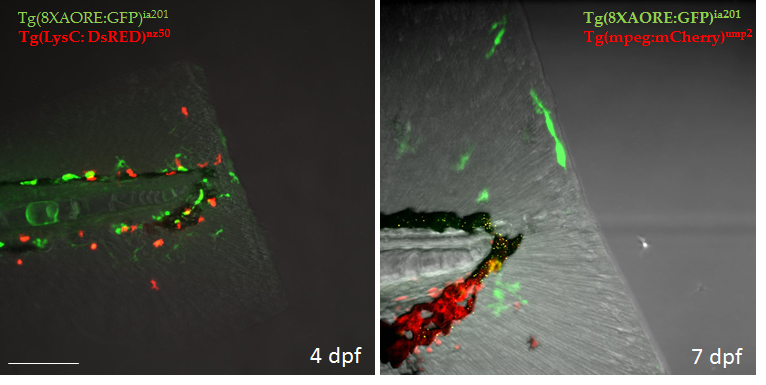

Supplement: Supplementary file 1 [file ijms-24-06804-s001.zip › Figure S6.tif]
